# A Robust *In Vivo*-Like Persistent Firing Supported by a Hybrid of Intracellular and Synaptic Mechanisms

**DOI:** 10.1371/journal.pone.0123799

**Published:** 2015-04-22

**Authors:** Arthur Jochems, Motoharu Yoshida

**Affiliations:** 1 International Graduate School of Neuroscience, Ruhr-University Bochum, Bochum, Germany; 2 Faculty of Psychology, Mercator Research Group—Structure of Memory, Ruhr-University Bochum, Bochum, Germany; Georgia State University, UNITED STATES

## Abstract

Persistent firing is believed to support short-term information retention in the brain. Established hypotheses make use of the recurrent synaptic connectivity to support persistent firing. However, this mechanism is known to suffer from a lack of robustness. On the other hand, persistent firing can be supported by an intrinsic cellular mechanism in multiple brain areas. However, the consequences of having both the intrinsic and the synaptic mechanisms (a hybrid model) on persistent firing remain largely unknown. The goal of this study is to investigate whether a hybrid neural network model with these two mechanisms has advantages over a conventional recurrent network based model. Our computer simulations were based on in vitro recordings obtained from hippocampal CA3 pyramidal cells under cholinergic receptor activation. Calcium activated non-specific cationic (CAN) current supported persistent firing in the Hodgkin-Huxley style cellular models. Our results suggest that the hybrid model supports persistent firing within a physiological frequency range over a wide range of different parameters, eliminating parameter sensitivity issues generally recognized in network based persistent firing. In addition, persistent firing in the hybrid model is substantially more robust against distracting inputs, can coexist with theta frequency oscillations, and supports pattern completion.

## Introduction

Behavioral studies indicate involvement of the prefrontal cortex and medial temporal lobe (MTL) during memory tasks that require short-term (200ms 2013 30s) information retention [1–4]. Electrophyiological recordings during these memory tasks indicate that persistent firing (a continuous firing of subset of neurons for the duration of the memory maintenance) may underly short-term information retention in the hippocampus [5,6], entorhinal cortex [7] and prefrontal cortex [8–11]. Cholinergic receptor activation is crucial in tasks that require short-term information retention [12–14].

As for the mechanism underlying persistent firing, it has been hypothesized that recurrent synaptic connectivity allows neural networks to support persistent firing [15–20]. However, models that utilize recurrent synaptic connectivity to drive persistent firing suffer from hyper- and hypo excitability. Early models that addressed this problem approximated neurons as linear elements [21–23]. These models tried to regulate the firing frequency of the persistent activity by setting the synaptic connections of the network so that inputs to each cell equaled the output of each cell [21–23]. However, these models were sensitive to small perturbations [24]. To address this problem, additional mechanisms such as the temporal synaptic dynamics [25,26] and feedback inhibition [15] have been proposed.

On the other hand, in vitro studies have long been indicating that persistent firing can be supported by individual neurons under cholinergic receptor activation supported by the calcium activated non-selective cationic (CAN) current [27–29]. We have recently shown that the CAN current supports persistent firing in the hippocampal CA3 area where recurrent synaptic mechanism has been proposed to support persistent firing [30]. Although hybrid of network and single cell mechanisms has been tested in firing rate models [31,32], the role of the CAN current has not been fully explored in spiking neural network model with recurrent synaptic connections [33–35]. To date, fundamental questions still remain to be elucidated: Can the hybrid model 1) support persistent firing with a frequency observed in vivo, 2) allow persistent firing to co-exist with theta oscillations, 3) reduce parameter sensitivity of persistent firing, and 4) support pattern completion? In this study, we addressed these using neural network models with conductance based spiking neuron models which are carefully tuned to mimic the in vitro recordings.

## Methods

All simulations were conducted using the NEURON simulation environment [36].

### Pyramidal cell model

Single compartmental Hodgkin-Huxley style pyramidal cells model was built based on the work by Pospischil and colleagues [37]. The kinetics of the CAN current was based on the model by Destexhe and colleagues [38] and was modified to fit our electrophysiological recordings.

The membrane potential of each pyramidal cell is described by

$$C_{m}\frac{dV}{dt}=-g_{L}\left(V-E_{L}\right)-I_{Na}-I_{K}-I_{M}-I_{Ca}-I_{CAN}-I_{syn}$$

where V is the membrane potential of the cell and *C*_*m*_ denotes the capacitance of the membrane (1 μF/cm^2^). *E*_*L*_ is the reversal potential for the leak current (-80 mV). *g*_*L*_ is the conductance of the leak channels (0.01 mS/cm^2^). *I*_*Na*_ and *I*_*K*_ are the fast sodium and potassium currents that are responsible for the generation of action potentials. *I*_*M*_ is the M-current. *I*_*Ca*_ is a high threshold calcium current. *I*_*CAN*_ is the CAN current. *I*_*syn*_ is the current that results from synaptic input to the cell.

#### Kinetics of ionic currents

The sodium current is described by the following equations:

$$\begin{array}{l} I_{Na}={\bar{g}}_{Na}m^{3}h\left(V-E_{Na}\right)\\ \frac{dm}{dt}=\alpha_{m}\left(V\right)\left(1-m\right)-\beta_{q}\left(V\right)m\\ \frac{dh}{dt}=\alpha_{h}\left(V\right)\left(1-h\right)-\beta_{h}\left(V\right)h\\ \alpha_{m}=\frac{-0.32\left(V-13\right)}{e^{\left(-\frac{v-13}{4}\right)}-1}\\ \beta_{m}=\frac{0.28\left(V-40\right)}{e^{\left(\frac{v-40}{5}\right)}-1}\\ \alpha_{h}=0.128e^{\left(\frac{V-17}{18}\right)}\\ \beta_{h}=\frac{4}{1+e^{\left(-\frac{V-40}{5}\right)}} \end{array}$$

where ${\bar{g}}_{Na}$ = 50 mS/cm^2^ and *E*_*Na*_ = 50 mV.

The potassium current is described by the following equations:

$$I_{K}={\bar{g}}_{K}n^{4}\left(V-E_{K}\right)$$

where ${\bar{g}}_{K}$ = 5 mS/cm^2^ and *E*_*K*_ = -100 mV.

The M-current is described by the following equations:

$$\begin{array}{l} I_{\text{M}}=\;{\bar{g}}_{M}p\left(V-E_{K}\right)\\ \frac{dp}{dt}=\left(p_{\infty }\left(V\right)-\;p\right)/\tau_{p}\left(V\right)\\ p_{\infty }\left(V\right)=\;\frac{1}{1+\;e^{\left(-\left(v+35\right)/10\right)}}\\ \tau_{p}\left(V\right)=\;\frac{\tau_{max}}{3,3e^{\left(\frac{V+35}{20}\right)}+e^{-\left(\;\frac{V+35}{20}\right)}} \end{array}$$

where ${\bar{g}}_{M}$ = 30 μS/cm^2^ and *τ*_*max*_ = 4s.

The voltage dependent high-threshold calcium current is described by the following equations:

$$\begin{array}{l} I_{Ca}=\;{\bar{g}}_{Ca}q^{2}r\left(V-E_{Ca}\right)\\ E_{Ca}=\frac{T}{2F}\text{log}\frac{{\left[ca\right]}_{o}}{{\left[ca\right]}_{i}}\\ \frac{dq}{dt}=\alpha_{q}\left(V\right)\left(1-q\right)-\beta_{q}\left(V\right)q\\ \frac{dr}{dt}=\alpha_{r}\left(V\right)\left(1-r\right)-\beta_{r}\left(V\right)r\\ \alpha_{q}=\;\frac{0.055\;\left(-27-V\right)\;}{e^{\left(\frac{-27-V}{3.8}\right)}-1}\\ \beta_{q}=\;0.94e^{\left(\frac{-75-V}{17}\right)}\\ \alpha_{r}=\;0.000457\;e^{\left(\frac{-13-V}{50}\right)}\\ \beta_{r}=\;\frac{0.0065\;}{e^{\left(\frac{-15-V}{28}\right)}+1} \end{array}$$

where ${\bar{g}}_{Ca}$ = 0.1 mS/cm^2^, T represents the temperature in Kelvin. F represents the faraday constant.

The CAN current is described by the following equations:

$$\begin{array}{l} I_{CAN}=\;{\bar{g}}_{CAN}m^{2}\left(V-E_{CAN}\right)\\ \frac{dm}{dt}=\left(m_{\infty }\left({\left[Ca\right]}_{i}\right)-\;m\right)/\tau_{m}\left({\left[Ca\right]}_{i}\right)\\ m_{\infty }\left({\left[Ca\right]}_{i}V\right)=\;\frac{\alpha \left({\left[Ca\right]}_{i}\right)}{\alpha \left({\left[Ca\right]}_{i}\right)+\;\beta }\\ \tau_{m}\left({\left[Ca\right]}_{i}\right)=\;\frac{\tau_{adj}}{\alpha +\beta }\\ \tau_{adj}=\;3^{\left(\frac{T\;-22}{10}\right)}\\ \alpha \left({\left[Ca\right]}_{i}\right)=\;\beta +\;{\left(\frac{{\left[Ca\right]}_{i}}{{\left[Ca\right]}_{c}}\right)}^{2} \end{array}$$

where ${\bar{g}}_{CAN}$ ^2^, the maximal conductance of the CAN current, was changed between 0 to 8.67 μS/cm^2^, depending on the simulation. *E*_*CAN*_, the potential of the CAN current, was -20 mV. *τ*_*adj*_, is a constant that takes temperature changes into account. In our simulations, the temperature was always set to 36°C. *β* was 0.002 ms^-1^.

Biological mechanism of CAN current activation involves G-protein coupled receptors. Under the cholinergic tone, a muscarinic acetylcholine receptor (mAchR) activation leads to an activation of phospholipase C (PLC) and a breakdown of phosphatidylinositol 4,5-bisphosphate (PIP2). Both of these are involved in the activation of the CAN current [39]. To mimic heightened cholinergic tone throughout memory task, this effect was modeled simply by setting the value of ${\bar{g}}_{CAN}$ to a higher value.

In addition, calcium plays an important role [40,41] where a rise of intracellular calcium concentrations to a certain level activates the CAN current [42]. Since intracellular calcium concentration changes dynamically dependent on neural activity, this is modeled explicitly by linking the CAN current gate valuable m with the intracellular calcium concentration ([*Ca*]_*i*_).

[*Ca*]_*c*_ is a scaling constant experimentally chosen to fit our experimental recordings of persistent firing, and was set to 0.75 μM. This parameter was modified from the original value (1 μM) described by Destexhe and colleagues [38]. First, the value of [*Ca*]_*c*_ controls the number of spikes required to induce persistent firing. This is demonstrated in S1A and S1B Fig. A brief but relatively strong current injection (500 pA, 86 ms) was sufficient to induce persistent firing when [*Ca*]_*c*_ value was 0.65 μM (S1A Fig) but not when it was 0.85 μM (S1B Fig). This is because the calcium increase did not activate sufficient CAN current to drive persistent firing when [*Ca*]_*c*_ value was 0.85 μM. Second, the value of [*Ca*]_*c*_ also controlled the frequency and maintenance of persistent firing as shown in S1C–S1E Fig. In these simulations, much longer current stimulation (2s, 150pA) was used to make sure the calcium concentration rises sufficiently to initiate persistent firing. Increasing the value of [*Ca*]_*c*_ caused a reduction in persistent firing frequency (compare S1C and S1D Fig) and eventual cessation of persistent activity (S1E Fig). The [*Ca*]_*c*_ value was set to 0.75 μM for further simulations in this paper because several spikes were sufficient to induce persistent firing as observed in vitro, and the observed persistent firing frequency (5.2 Hz) was well within the range of observed persistent firing frequencies during our in-vitro recordings (3.5 to 17.5 Hz).

In addition, we confirmed that our CAN current model replicates voltage clamp recordings from the hippocampus where a transient activation of the CAN current was triggered by single pulse stimulation [43]. The behavior of the CAN current in our model in voltage clamp is shown in S2 Fig. This behavior is also similar to the voltage clamp recording from entorhinal cells [44] which shows similar persistent firing due to the CAN current. In summary, original CAN current model by Destexhe and colleagues [38] were tuned based on both current clamp and voltage clamp recordings from hippocampal cells, making our CAN current model a good estimation of the hippocampal CAN current.

#### Submembranal Ca^2+^ concentration

The dynamics of intracellular submembranal calcium concentration was modeled as follows based on Destexhe and colleagues [38]:

$$\begin{array}{l} \frac{d{\left[Ca\right]}_{i}}{dt}=\;\gamma \left({\left[Ca\right]}_{i}\right)+\;\frac{\left({\left[Ca\right]}_{\infty }-\;{\left[Ca\right]}_{i}\right)}{\tau_{r}}\\ \gamma \left({\left[Ca\right]}_{i}\right)=\frac{-k{\left[Ca\right]}_{i}}{2Fd} \end{array}$$

In which [*Ca*]_∞_ is a constant that represents the amount of calcium inside of the cell in resting condition. In our simulations, a value of 200 nM is used. *τ*_*r*_ is a constant used to determine the rate of calcium removal from the cell (1 s). The variable k is used as a units conversion constant, and was set to 10^5^. F represents the faraday constant, 96489 coulomb. Finally, the value d represents the depth of a shell in which the calcium is stored (1μm).

### Interneuron model

In order to simulate interneurons, we have adopted the fast spiking interneuron model from Pospischil and colleagues [37]. The electrophysiological properties of this neuron resemble those of aspiny inhibitory neurons [37]. This model contains only the leak, sodium and potassium currents. The membrane potential of an inhibitory neuron can be described by

$$C_{m}\frac{dV}{dt}=-g_{L}\left(V-E_{L}\right)-I_{Na}-I_{k}$$

In which *I*_*Na*_ *and I*_*k*_ are the sodium and potassium currents as described earlier. The equilibruim potental for the leak current *E*_*L*_ was -70mV. The equilibium potential for the sodium current *E*_*na*_ was 50 mV. The equilibium potential for the potassium current *E*_*K*_ was -100mV. The conductance of the leak current *g*_*L*_ was 0.15 mS/cm^2^. The maximum conductance of the sodium current ${\bar{g}}_{\text{N}a}\;$ was 50 mS/cm^2^ and the maximum conductance of the potassium current ${\bar{g}}_{K}$ was 10 mS/cm^2^

### Synaptic model

Synaptic transmission is modeled using a double exponential function

$$I_{syn}=w\left(e^{-\frac{t}{\tau_{1}}}-e^{-\frac{t}{\tau_{2}}}\right)$$

where *w* is the synaptic weight, *τ*_1_ is the rise time constant and *τ*_2_ is the decay time constant of the synapse. For AMPA synapses, a rise time of 0.5 ms and a decay time of 2.4 ms were chosen in agreement with Symes and Wennekers [45]. For GABAergic synapses, a rise time of 1 ms and a decay time constant of 7 ms was used [45]. Synaptic delay was set to 2 ms based on physiological observations (~1–3 ms; [46, 47]).

## Results

### Response of single pyramidal cell model

**Fig 1:**
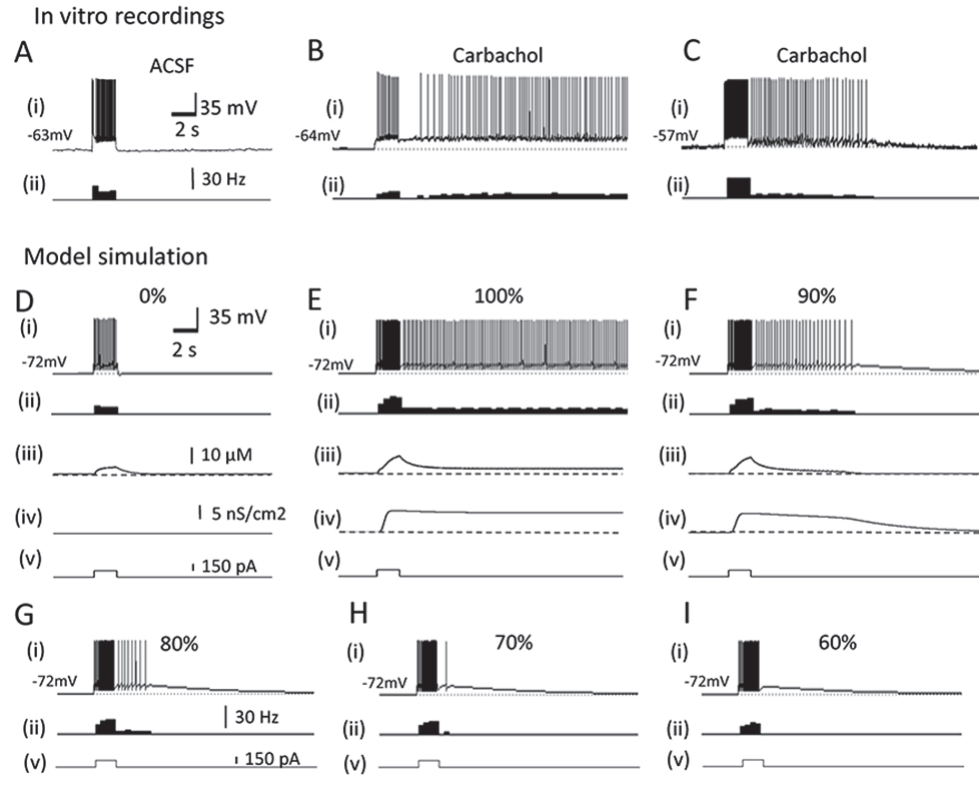
The CAN current supports persistent firing in the single cell model. (A) Response of an in vitro hippocampal CA3 pyramidal cell in the normal ACSF. The brief current injection did not induce persistent firing. Ionotropic synaptic blockers, 20 μM CNQX, 50 μM D,L-APV and 100 μM PTX, were present in all in vitro recordings to block AMPA/kainite receptors, NMDA receptors and GABA_A_ receptors, respectively. (B) Response of the same cell in carbachol (10 μM). Long-lasting (≥ 30 s) persistent firing is observed after the same brief stimulation. (C) Self-terminating persistent firing (< 30 s) was observed in the same condition as in B in some cells. (D) Response of the single pyramidal cell model to a brief current injection without the CAN current. No persistent firing was seen. (E) Long-lasting persistent firing observed in the single pyramidal cell model with 100% CAN current. (F) Self-terminating persistent firing observed in the single pyramidal cell model with 90% CAN current. (G-I) Further reduction of the CAN current caused self-terminating persistent firing with shorter durations (G and H) and an after-depolarization without spikes (I). In all figures, (i) Membrane potential, (ii) Frequency of firing, (iii) Intracellular calcium concentration, (iv) CAN current conductance, and (v) Current injection.

We have recently reported that pyramidal cells in the hippocampal CA3 area support persistent firing through an intrinsic cellular mechanism in the presence of a cholinergic agonist carbachol [30]. An induction of persistent firing was tested by a brief current injection (100 pA, 200 ms—2 s). In the normal artificial cerebrospinal fluid (ACSF), pyramidal cells did not respond with persistent firing (Fig 1A). In the presence of carbachol (10 μM), we observed persistent firing. Majority of cells showed persistent firing which lasted more than 30 s and did not stop by itself (Fig 1B). We called this type of persistent firing a long-lasting persistent firing and it continued until the experimenter turned it off by injecting a negative current. The frequency of persistent firing ranged from 3.5 to 17.5 Hz. Other cells responded with self-terminating persistent firing which lasted for less than 30 s in the same condition (Fig 1C). These recordings were performed in the presence of ionotropic synaptic blockers, 20 μM CNQX, 50 μM D,L-APV and 100 μM PTX, to block AMPA/kainite receptors, NMDA receptors and GABA_A_ receptors, respectively. Similar long-lasting and self-terminating persistent firing have been observed in multiple areas in the MTL [39, 48, 49]. This persistent firing has been shown to be driven by the CAN current which is intrinsic to individual neurons [39, 48, 49].

Fig 1D–1F show the responses of the pyramidal cell model to a similar stimulation. When the CAN current was not included (g_CAN = 0), current injection (150 pA, 2s) did not induce persistent firing (Fig 1D). This condition mimics in vitro recordings with the normal ACSF without carbachol (Fig 1A). In contrast, the same stimulation induced a long-lasting persistent firing when we included the CAN current (g_CAN = 8.67μS/cm^2^; Fig 1E). This condition mimics the carbachol condition in vitro (Fig 1B). As illustrated in Fig 1E(iii), intracellular calcium concentration increased during the current injection as the cell elicited spikes. This triggered an activation of the CAN current as shown in Fig 1E(iv), and this CAN current activation supported long-lasting persistent firing. At this value of g_CAN, the frequency of persistent firing was 5.2 Hz. By reducing g_CAN to 90–70% of this value, the model responded with self-terminating persistent firing with various durations (Fig 1F–1H). This suggests that the cells which responded with self-terminating persistent firing in vitro (Fig 1C) might have had relatively small amount of CAN current available. When the CAN current was reduced to 60% of the original value, only an afterdepolarisation without spike was observed (Fig 1I).

These results showed that the pyramidal cell model replicates both long-lasting and self-terminating persistent firing as observed in vitro.

### The hybrid model supports persistent firing within physiological frequency in a wide range of synaptic conductance

**Fig 2:**
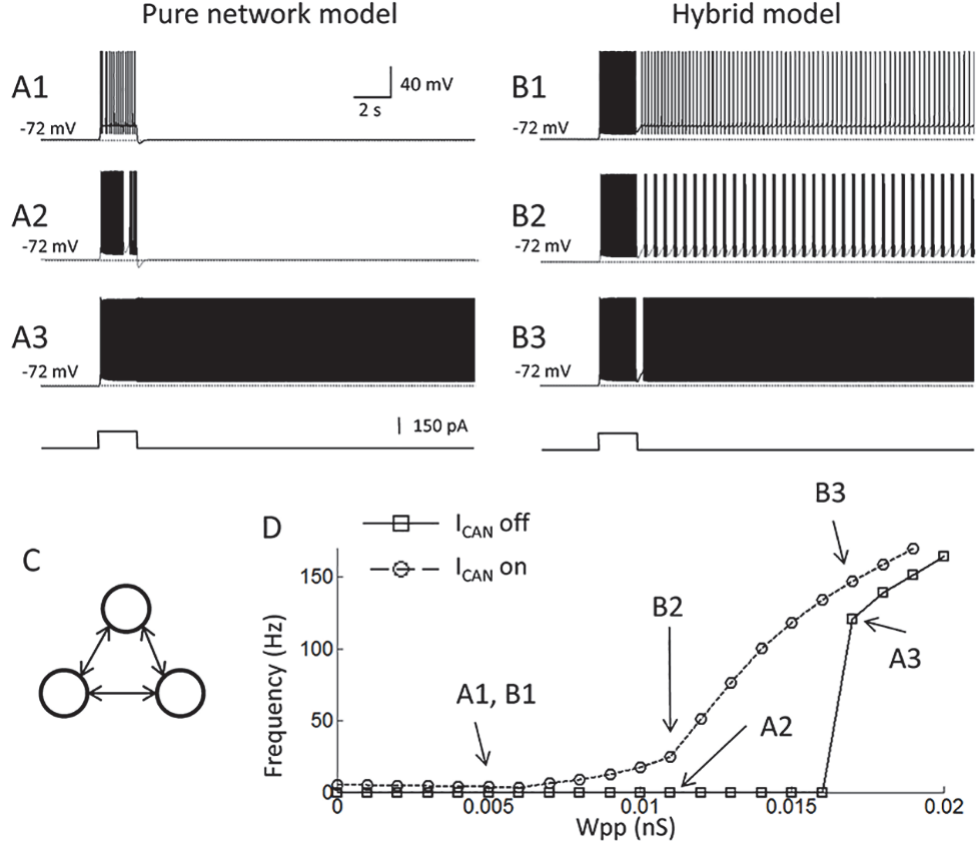
The hybrid model supports persistent firing within a physiological frequency range across a wide range of synaptic conductances. (A1-3) Responses of the pure network model to a brief stimulation. Conductance of recurrent synaptic connections (Wpp) was 0.005, 0.011, and 0.017 nS, respectively. Activity of only one of the pyramidal cell models is shown. Bottom trace indicates current injection. (B1-3) Responses of the hybrid model with Wpp = 0.005, 0.011 and 0.017 nS, respectively. Frequency of persistent firing increased gradually with the increase in Wpp. (C) Model network structure. Three pyramidal cells were connected in an all-to-all fashion. (D) Frequency of persistent firing as a function of the synaptic conductance (Wpp). While pure network model (solid line with square symbols, I_CAN_ off) shows an abrupt frequency jump at around Wpp = 0.016 nS, the hybrid model (dotted lines with circle symbols, I_CAN_ on) shows a gradual increase of the frequency as a function of Wpp covering frequency range observed in vivo (3–50 Hz). Letters in the figure correspond to Figs. A1-3 and B1-3. I_CAN_: CAN current.

As mentioned above, persistent firing driven solely by an excitatory recurrent synaptic connectivity may critically depend on the synaptic conductance between cells in the network. Too little excitation will cause the activity to run down, resulting in the absence of persistent firing, while too much excitation will cause the network to become overexcited [50]. To compare the sensitivity of persistent firing to the synaptic conductance (Wpp) in a pure network model and a model with the CAN current, we set up a simple neural network with three pyramidal cell models which were connected in an all-to-all fashion (Fig 2C).

First, we investigated the frequency of persistent firing without the CAN current (g_CAN = 0). A current injection (150 pA, 2 s) was given to all three neurons and the frequency of persistent firing was measured at 10–20 s after the offset of the current injection. Fig 2A1 shows the firing pattern of one of the three neurons in the network when Wpp was 0.005 nS. Since the network was symmetric, other two neurons behaved exactly the same in this simulation. Persistent firing was not observed in this case or when Wpp was increased to 0.011 nS (Fig 2A2). Persistent firing occurred when Wpp was strengthened to 0.017 (Fig 2A3). At this value of Wpp, the frequency of persistent firing was 120.4 Hz. Fig 2D summarizes the frequency of persistent firing as a function of synaptic connection (Wpp). In this simple model without the CAN current, persistent firing with relatively high frequency (> 100 Hz) abruptly occurred above Wpp = 0.016 nS (solid line with square symbols). Therefore, this model did not support the frequency of persistent firing often observed in vivo (3–50Hz; [3,5,6,51–55]).

As the next step, we tested persistent firing in the same network model with the CAN current in all pyramidal cells (the hybrid model; g_CAN = 8.67μS/cm^2^). With this amount of CAN current, persistent firing was supported with all values of Wpp tested (Fig 2B1–2B3). However, in contrast to the pure-network based persistent firing, the frequency of persistent firing in this model gradually increased as a function of Wpp (Fig 2D; dotted lines with circules). The frequency of persistent firing was within the range observed in vivo (3–50 Hz) in a wide range of Wpp (0–0.012 nS).

**Fig 3:**
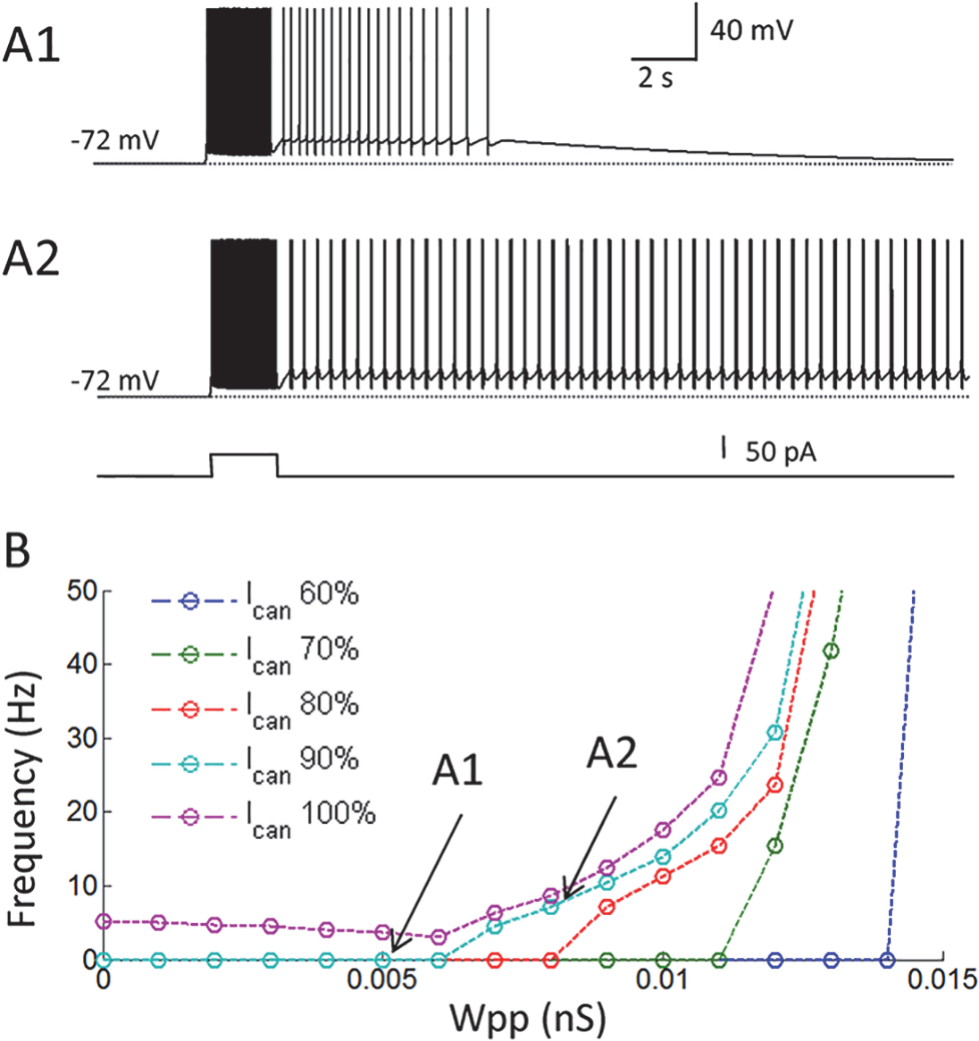
Persistent firing with smaller CAN current conductances. (A1 and 2) Responses of the model with 90% CAN current conductance. Conductance of recurrent synaptic connections (Wpp) was 0.005 and 0.008 nS, respectively. (B) Frequency of persistent firing as a function of Wpp. Relatively small CAN current conductances, which supported only self-terminating persistent firing in the single cell model, supported long-lasting persistent firing in the network model. A gradual increase of firing frequency was observed with these smaller CAN current conductances as well. I_CAN_: CAN current.

We also tested persistent firing with smaller CAN current conductances (Fig 3). As shown in Fig 1, self-terminating persistent firing was observed when the CAN current conductance was reduced to 70–90%. Fig 3A1 and 3A2 show the persistent firing of one of the three cells in the network with 80% CAN current conductance. When the synaptic conductance (Wpp) was 0.005 nS, the network model supported self-terminating persistent firing (Fig 3A1). However, when Wpp was increased to 0.008 nS, the network supported long-lasting persistent firing (Fig 3A2). Fig 3B shows the frequency of persistent firing as a function of Wpp with different levels of CAN current conductance. These results show that lower CAN current conductances which support only self-terminating persistent firing in individual cells (70–90%), can support long-lasting persistent firing in a network within the in vivo frequency range. However, when the conductance of the CAN current is reduced to a level in which no self-terminating persistent firing is supported in the single cell level (< 60%), the network could not support persistent firing in the in vivo frequency range.

In summary, these results suggest that the hybrid model with the CAN current may support persistent firing with a frequency observed in vivo (3–50 Hz) with a wide range of synaptic conductance. In addition, relatively low levels of the CAN current, which support only self-terminating persistent firing in individual cells, are also sufficient to support long-lasting persistent activity with a frequency observed in vivo (3–50 Hz) with a wide range of synaptic conductances.

### Persistent firing in the hybrid model is more robust against distracting stimuli

**Fig 4:**
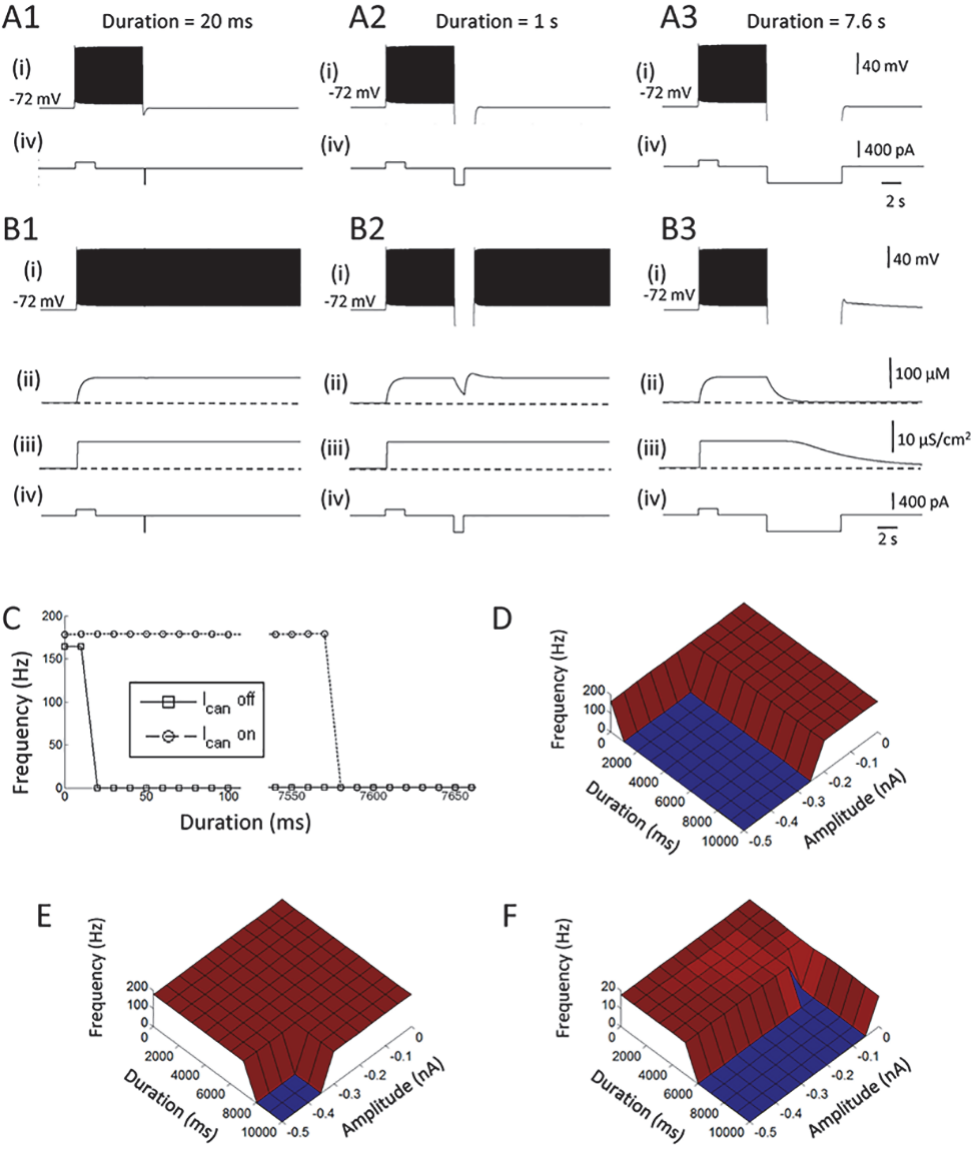
Persistent firing in the hybrid model is more robust against distracting stimuli. (A1-3) Responses of the pure network model to a negative current injection with different durations: 20 ms, 1 s, and 7.6 s, respectively. (B1-3) Responses of the hybrid network model to a negative current injection with different durations: 20 ms, 1 s, and 7.6 s, respectively. (C) Frequency of persistent firing as a function of the duration of the negative current injection. Persistent firing in the pure network model was easily terminated by a short (≥ 20 ms) current injection. (D) Frequency of persistent firing in the pure network model as a function of the duration and the amplitude of the negative current injection. (E) Same as in D from the hybrid network model. Persistent firing is more robust in the hybrid model. (F) Same as in E but with a reduced Wpp (0.01 μS). Note different frequency scale. In all figures, (i) Membrane potential, (ii) Intracellular calcium concentration, (iii) CAN current conductance, and (iv) Current injection. I_CAN_: CAN current.

As the next step, we tested whether the CAN current contributes to the robustness of persistent firing. First, we tested the robustness of persistent firing in the pure network model without the CAN current. For this simulation, the synaptic conductance (Wpp) was set to 0.02 nS because this Wpp supported persistent firing both in models with and without the CAN current. Persistent firing was initiated by the same brief stimulation (150 pA, 2 s; Fig 4A1). Subsequently, a negative but very brief current (-400 pA, 20 ms) was injected to all cells 5 s after the initiation of persistent firing. This negative current turned off the persistent firing in the pure network model (Fig 4A1). Fig 4A2 and 4A3 shows simulations with similar current injections with longer durations (1s and 7.6 s, respectively). These longer current injections also terminated persistent firing in the pure network model.

On the other hand, persistent firing in the hybrid model with the CAN current was not terminated when the duration of the stimulation was 20 ms and 1s (Fig 4B1 and 4B2, respectively). Persistent firing with the CAN current was, however, terminated when the duration of stimulation was 7.6 s (Fig 4B3). This robustness is due to relatively slow deactivation of the CAN current (Fig 4B3(iii); [27,56]). As summarized in Fig 4C, persistent firing in the model with the CAN current was much more robust to hyperpolarizing stimulation compared to the pure network model. However, it was possible to turn off persistent firing in the network with the CAN current when a long duration current injection was used.

Second, we investigated the effect of negative current injections varying both the duration and the amplitudes of the current (Fig 4D and 4E). While the persistent firing in the pure network was terminated by a current injection larger than -250 pA and longer than 1 s (Fig 4D), persistent firing with the CAN current required a current injection larger than -350 pA and longer than 8 s (Fig 4E).

As shown in Fig 2D, the network model with the CAN current supports persistent firing in the physiological frequency range when Wpp was set to < 0.012 nS. Therefore, we tested the robustness of persistent firing within the physiological frequency range using Wpp = 0.01 nS (frequency = 17.6 Hz; Fig 4F; note different frequency scale than Fig 4D and 4E). This model with a smaller Wpp still required at least 5 s of hyperpolarization, which was substantially longer than that in the pure network model tested above (Fig 4D). On the other hand, this model with Wpp = 0.01 μS required much less current amplitude (0.05 nA) suggesting that more physiological levels of hyperpolarization is sufficient for turning off persistent firing within physiological frequency range.

### Persistent firing in the hybrid model can co-exist with theta rhythm

Theta rhythm is a hallmark of hippocampus during learning and spatial exploration in rodents [57]. Strong hippocampal theta oscillations are observed during working memory and short-term memory tasks in which persistent firing is observed [58,59]. However, it remains unclear whether persistent firing can co-exist with periodic oscillatory input. Therefore, we aimed to elucidate the effect of imposed oscillations on persistent activity using an injection of sinusoidal current to all three pyramidal cells in the network. The frequency of the oscillation was 7 Hz and the amplitude was modulated. Wpp was set to 0.018 nS and 0.008 nS in the simulations without and with the CAN current, respectively.

**Fig 5:**
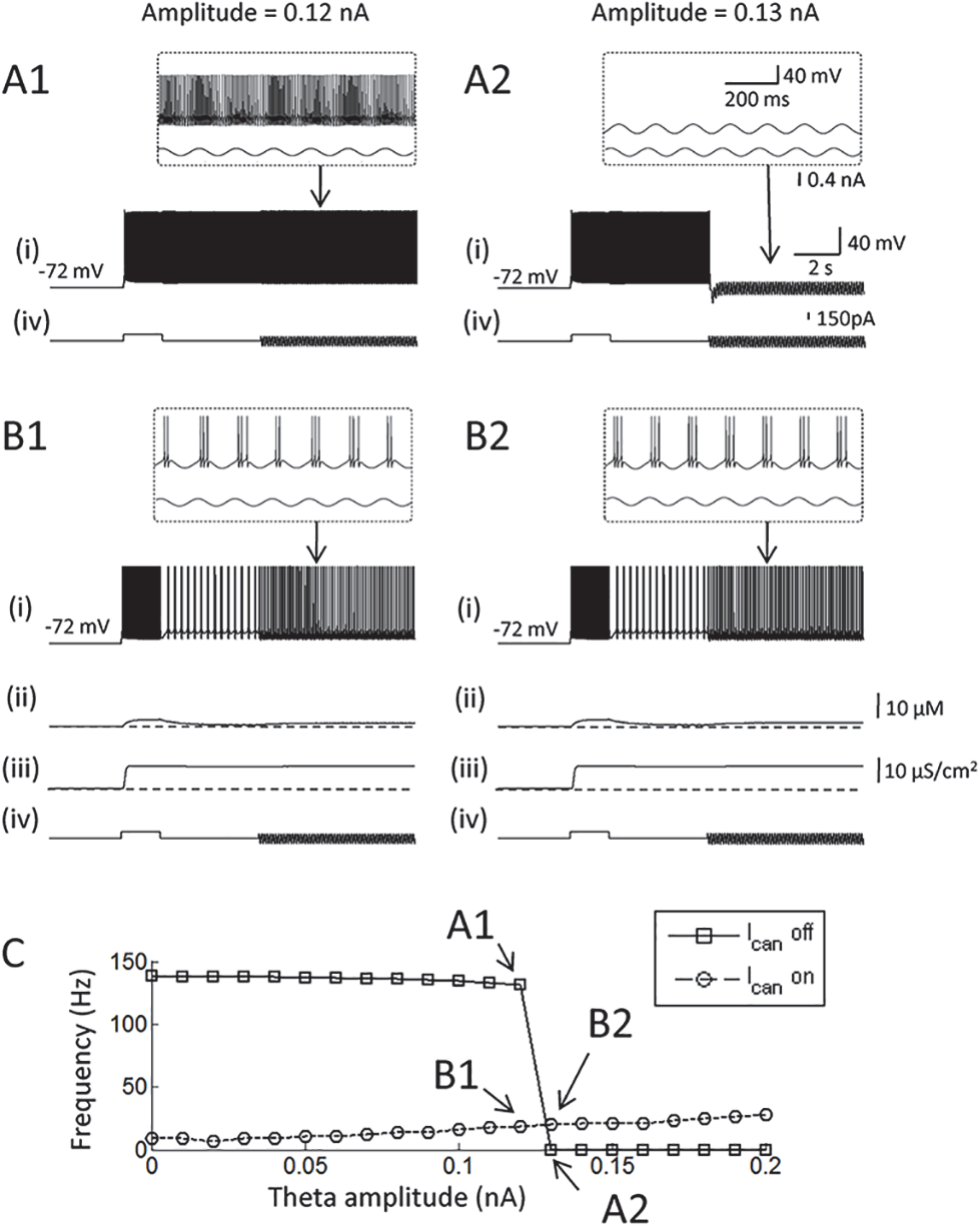
Persistent firing in the hybrid model can co-exist with theta rhythm. (A1 and 2) Responses of the pure network model to a 7 Hz sinusoidal current injection. The amplitude of the current injection was 0.12 nA in A1, and 0.13 nA in A2. (B1 and 2) Responses of the hybrid network model to the same current injection with an amplitude of 0.12 nA in B1, and 0.13 nA in B2. Theta entrainment was observed only in the hybrid model. (C) Frequency of persistent firing as a function of the amplitude of the sinusoidal current injection. In all figures, (i) Membrane potential, (ii) Intracellular calcium concentration, (iii) CAN current conductance, (iv) Current injection. I_CAN_: CAN current.

Fig 5A1 shows the activity of one of the three cells in a pure network model. The sinusoidal current injection with an amplitude of 0.12 nA started 5 s after the initiation of persistent firing (Fig 5A1 (iv)). Persistent firing continued during this sinusoidal current injection, and the frequency of persistent firing was weakly modulated by the imposed oscillation (Fig 5A1). This is seen as the dense and sparse firing at the peak and the trough of the theta frequency oscillations, respectively (Fig 5A1, inset). However, firing pattern of the cell was not perfectly entrained by the oscillations indicating that stronger sinusoidal current injection amplitude is needed for the cell to fire in a typical theta burst firing pattern. However, when the amplitude of the theta oscillation was increased to 0.13 nA, persistent firing was terminated by the sinusoidal current injection (Fig 5A2). This is because of suppressed firing at the trough of the oscillations. As shown in the previous section, discontinuation of firing as short as 20 ms is sufficient to terminate persistent firing in the pure network model. Therefore, suppressed firing at the trough during the first cycle of the sinusoidal current injection terminated the persistent firing in this case.

On the other hand, persistent firing continued during the injection of the sinusoidal current injection in the model with the CAN current despite suppressed firing at the trough of theta (Fig 5B1 and 5B2). This is due to the fact that persistent firing in the model with CAN current is much more robust to temporal absence of spiking as shown in the previous section. Fig 5C summarizes the effect of rhythmic input on persistent activity with variety of amplitudes. While sinusoidal current injections with amplitudes larger than 120 pA turn off persistent firing in pure network model, current injections have little effect on the persistent firing in the hybrid model with the CAN current.

These observations indicate that persistent firing in the hybrid model is more robust to inhibitory stimuli than in the pure network model, and this feature allows persistent firing in the hybrid model to co-exist with the intracellular theta oscillations.

### The effect of the CAN current in a network model with a feedback inhibition

**Fig 6:**
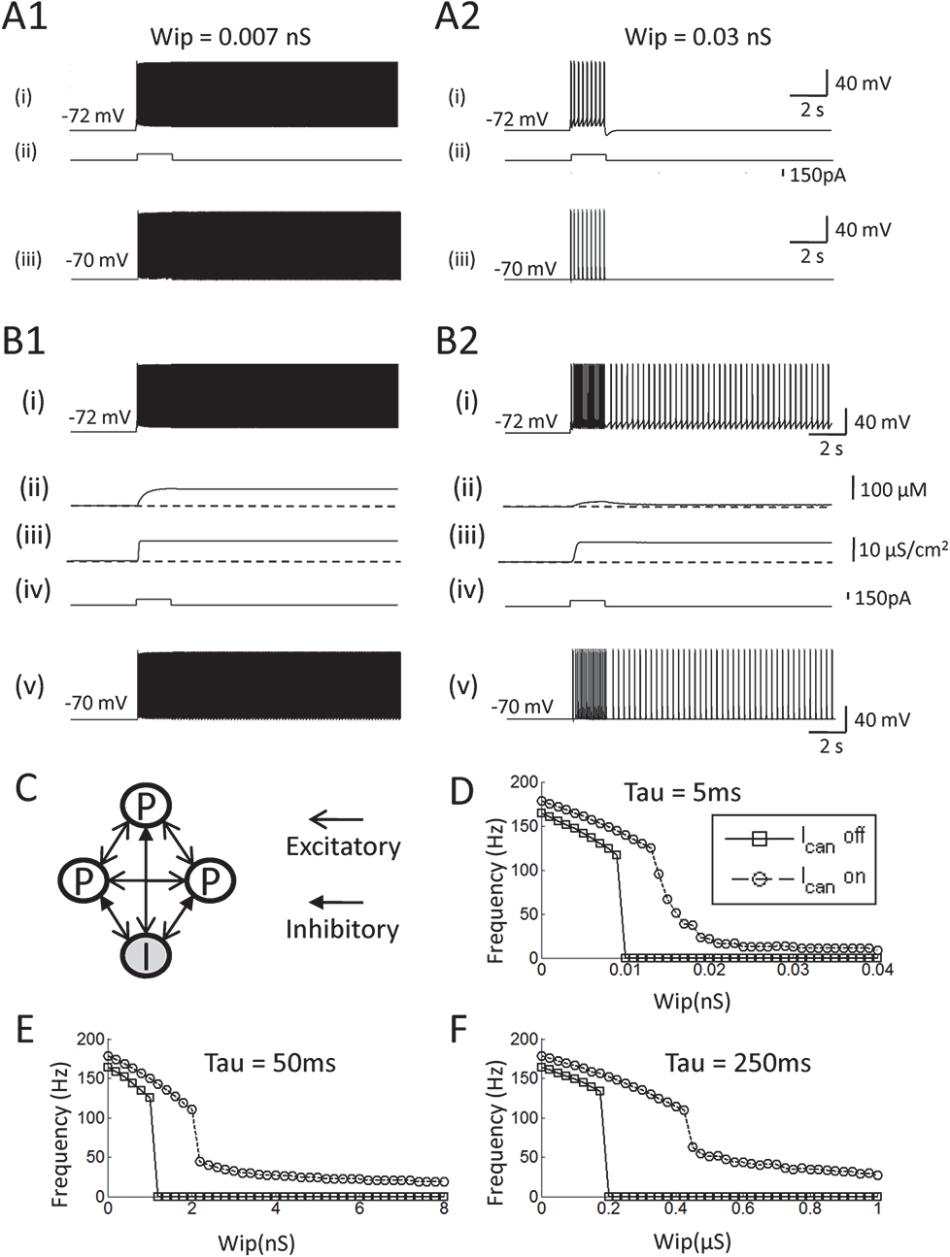
The effect of the CAN current in a network model with a feedback inhibition. (A1 and 2) Responses of the pure network model with different inhibitory synaptic conductances: 0.007 and 0.03 nS, respectively. (B1 and 2) Responses of the hybrid network model with different inhibitory synaptic conductances: 0.007 and 0.03 nS, respectively. (C) The model network structure. *White circles* represent pyramidal cells and *shaded circles represent* inhibitory cells. (D—F) Frequency of persistent firing as a function of the inhibitory synaptic conductance (Wip), with different time constants for IPSC: 5 ms, 50 ms and 250 ms, respectively. Frequency of persistent firing is modulated gradually by the increased inhibitory conductance in the hybrid model with the CAN current but not in the pure network model.

It has been shown that inhibitory synaptic feedback network can regulate the frequency of persistent firing to the physiological frequency range in a firing rate model [60]. Here, we tested the effect of feedback inhibition circuit for the frequency control in the pure network and the hybrid models. To achieve this goal, we added an inhibitory interneuron model to the three-cell network introduced above (Fig 6C). The inhibitory neuron received excitatory synaptic inputs from all three pyramidal cells and inhibited all three pyramidal cells. The inhibitory neuron model did not include the mechanism to support persistent firing consistent with our previous in vitro observation [30]. Wpp was 0.02 nS in these simulations as in the Section 3.3.

First, we examined the frequency of persistent firing in pyramidal cells with different levels of inhibitory feedback. In these simulations, the decaying time constant for the inhibitory synapses was set to 5 ms and the conductance of the inhibitory synapses from the inhibitory to the pyramidal cells was systematically changed. In the pure network model, persistent firing with a weak feedback inhibition (Wip = 0.007 nS) resulted in a high frequency persistent firing (> 100 Hz; Fig 6A1). When Wip was increased to 0.03 nS, persistent firing was not observed (Fig 6A2). As shown in Fig 6D, the frequency of persistent firing drops dramatically from over 100 Hz to 0 Hz (no persistent firing) around Wip = 0.01 nS.

In contrast, persistent firing in the model with the CAN current was modulated more gradually by the change of the inhibition strength. As the Wip was increased from 0.007 to 0.03 nS, the frequency of persistent firing was lowered to physiological frequency (12.8 Hz) instead of being completely terminated (Fig 6B1 and 6B2). As shown in Fig 6D, the frequency went down continuously as the Wip was increased, supporting the frequency range observed in vivo (3–50 Hz).

**Fig 7:**
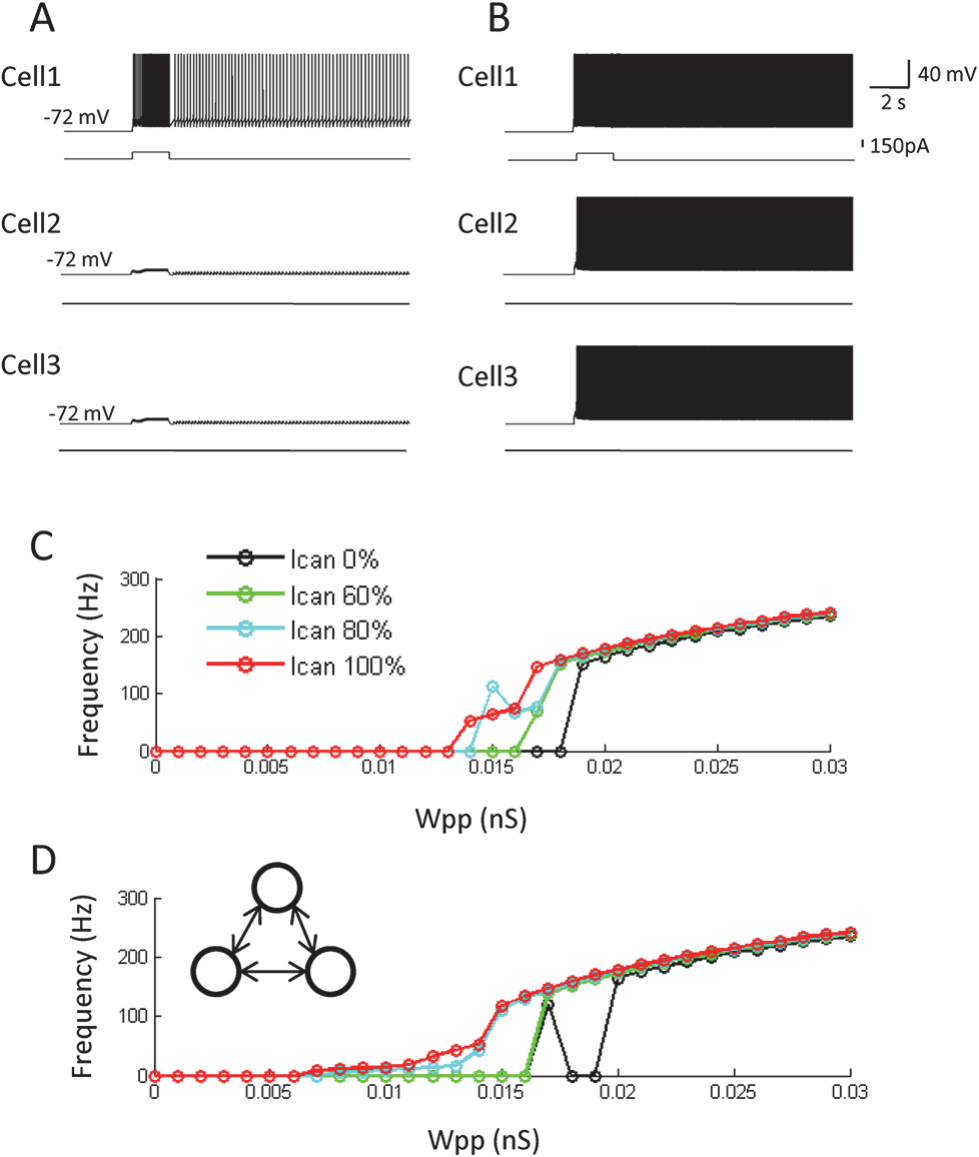
An ability for pattern completion is intact in the hybrid model. (A and B) Responses of all three cells in the hybrid model with different excitatory synaptic conductances: 0.005 nS and 0.015 nS, respectively. Note that the stimulation (retrieval cue) is given only to the cell 1. (C) Average frequency of persistent firing of the non-stimulated cells (cell 2 and cell 3) as a function of the synaptic conductance for different levels of CAN current conductance. (D) Frequency of persistent firing of the non-stimulated cells (cell 3) when stimulation was given to the cell 1 and cell 2 for different levels of CAN current conductance. The ability for pattern completion was present in the hybrid model within a physiologically observed frequency range, even when CAN current conductances were used that support only self-terminating persistent firing in individual cells.

In addition to the conductance of the inhibitory synapses, the time constant of these synapses may play an important role in the regulation of the firing frequency of persistent firing. Therefore, we studied the frequency of persistent firing with longer decay time constants (50 ms and 250 ms) for the inhibitory synapse (Fig 7E and 7F, respectively). In the pure network model, persistent firing still showed an abrupt frequency drop from > 100 Hz to 0 Hz. In contrast, persistent firing within the physiological range was observed for much wider range of Wip in the hybrid model with the CAN current (Fig 6E and 6F).

These results suggest that the simple form of inhibitory feedback tested here cannot easily regulate persistent firing frequency to the physiological frequency range in the pure network-based model. On the other hand, the frequency of persistent firing can be regulated to the physiological frequency using inhibitory feedback synapses in the model with the CAN current in a wide range of inhibitory synaptic conductance and time constant.

### Pattern completion

Studies report that the hippocampal CA3 area is involved in pattern completion [61,62]. Pattern completion is believed to be supported by recurrent synaptic connections [16,17,63,64]. In Marr's theory, memory was embedded as the strengthened recurrent connections among a group of neurons, and this strengthened network supported both persistent firing and pattern completion function [16]. Therefore, synaptic strengthening was correlated with pattern completion function. However, in our current study, we have demonstrated that persistent firing could be supported with a much wider range of recurrent synaptic conductance (Wpp) in the hybrid model (Figs 2 and 3). This relative insensitivity of persistent firing to synaptic conductance might weaken the causality between synaptic strengthening and pattern completion. Therefore, we tested whether pattern completion could still be performed in the model with the CAN current.

In order to test this, we first applied a stimulation (100 pA, 2 s) to one of the three neurons as a partial cue for retrieval, and tested whether this could initiate persistent firing in all three neurons (a retrieval of the completed pattern). Fig 7A and 7B both show activity of all three cells in the hybrid model with the CAN current. When Wpp was relatively weak (0.005 nS), only the stimulated neuron (Cell 1) showed persistent firing and pattern completion did not occur (Fig 7A). On the other hand, when stronger recurrent connections were used (Wpp = 0.015 nS), the same stimulation lead to persistent firing of all three cells (Fig 7B). Therefore, pattern completion can be supported in the model with the CAN current by changing the strength of recurrent connections.

Fig 7C shows the average firing frequency of two neurons which were not stimulated (Cell 2 and 3) after the stimulation to the other neuron for different levels of CAN current conductance. In the model with 100% CAN current conductance, the average frequency was zero when Wpp was lower than 0.013 nS. As the Wpp increased further, frequency went up gradually (Fig 7C, red). Therefore, simple form of pattern completion occurred in this model when Wpp ≥ 0.0135 nS. On the other hand, in the pure network model (0% CAN current), the switch from no-pattern completion to pattern completion abruptly occurred at Wpp = 0.0185 nS (Fig 7C, black). When the CAN current conductance was 60% and 80%, where single cells supported only self-terminating persistent firing, the results were somewhat in between 100% and 0% cases described above (Fig 7C, blue and green). Pattern completion occurred less abruptly with the increase in Wpp compared to the 0% case.

In addition, we tested the case where two neurons were stimulated. In this case, we measured the frequency of firing from the one remaining cell which was not stimulated. As shown in Fig 7D, pattern completion occurred above Wpp ≥ 0.007 nS in the model with the 100% CAN current, and the frequency increased gradually as the Wpp increased further. In the pure network model (0% CAN current), pattern completion occurred at Wpp = 0.017 nS and Wpp ≥ 0.02 nS (Fig 7D, black). The frequency of firing during pattern completion was over 100 Hz. When intermediate levels of CAN current conductance were tested, 80% CAN current conductance could support gradual increase in firing frequency but 60% CAN current conductance was not sufficient in doing so (Fig 7D, blue and green).

These results indicate that pattern completion can be supported both in the model with and without the CAN current, and the transition from non-memory to memory state is more gradual in the model with sufficient CAN current than the pure network model.

## Discussion

In this study, we investigated the consequences of having both intrinsic cellular properties and a recurrent neural network to support persistent firing. Recent identifications of the CAN current as the ionic mechanism supporting persistent firing in many brain areas, made it important to study the role of this specific mechanism in neural networks to understand its possible role in vivo. First, we found that the CAN current enables neural network to support persistent firing with relatively low frequency range observed in vivo (3–50 Hz) while the same network without the CAN current only supported persistent firing higher than 100 Hz. Second, relatively small CAN current conductance, which supported only self-terminating (duration < 30 s) persistent firing in individual cells, were sufficient in supporting long-lasting persistent firing in the physiological firing frequency range. Third, we found that pure network driven persistent firing was subject to a brief (~ 15 ms) hyperpolarising stimulus, while persistent firing driven by the CAN current was robust against destructive signal. This robustness enabled persistent firing in the hybrid model to coexist with theta frequency oscillations, while persistent firing in the pure network model could not. Fourth, we demonstrated that feedback inhibitory network was able to regulate the frequency of the persistent firing to the physiological range in the hybrid model, but not in the pure network model. Furthermore, we demonstrated that the hybrid model could support pattern completion as in the case with the pure network model. These results suggest that the CAN current makes persistent firing in neural networks more robust by reducing tuning sensitivity issues that are known in network driven persistent firing. The observations that theta oscillations can coexist with the CAN current driven persistent firing, and that pattern completion is still supported by the hybrid model, further indicate the relevance of the CAN current in in vivo mnemonic functions.

### Comparison to other models

Cellular bistability was combined with synaptic network using firing rate neuron models [31,32]. Both of these studies focused on the spatial stability of the "bump" activity in the prefrontal cortex, and issues explored in our current study were not investigated. In addition, the type of bistability used in those studies (conditional type) was different from the bistability supported by the CAN current in in vitro studies (unconditional type; [48, 49]).

Fransén and colleagues [65] simulated the behavior of neurons from the entorhinal cortex during a delayed match to sample task. Multi-compartmental Hodgkin-Huxley type neurons that included calcium dynamics and the CAN current were used to support persistent activity. It was found that cells in the network could support match enhancement activity as observed in in vivo studies [7,66]. Their main focus was to model the neural activity observed in the specific memory task, and they did not address the difference between persistent firing supported by networks with and without the CAN current. Among the comparisons we tested between the model with and without the CAN current, Fransén and colleagues [65] have also shown that the CAN current allows persistent firing to continue in the face of hyperpolarizing stimuli (less than 3 s). This observation is in agreement with our results which indicated relative robustness of the persistent firing supported by the CAN current to distracting stimuli.

Several models that used gamma oscillations and theta oscillations to support a multi-item working memory have been proposed [67–70]. In these models, a group of cells that represents a chunk of information fired each theta cycle, but only in a given gamma subcycle [68]. In addition to recurrent synaptic connectivity, these models used a CAN current driven spike afterdepolarisation to drive persistent activity [67–70]. The persistent activity in these models was phase locked to theta oscillations. Therefore, although these studies did not systematically compare the ability of theta modulated firing in a model with and without the CAN current, their results are in agreement with our observation that persistent firing with the CAN current can co-exist with the theta frequency oscillations.

Sidiropoulou and Poirazi (2012) studied stimulus selectivity of PFC neurons during working memory tasks. They developed multi compartmental Hodgkin-Huxley type models with a CAN current kinetics scheme. A combination of the CAN current and small scale network was also tested by the same group [34]. They have indicated that the existence of the CAN current aid persistent firing by reducing the requirement of the NMDA synaptic component. However, their focus was not entirely on the CAN current and many of the properties tested in our current study were not investigated.

In summary, to our knowledge, systematic comparisons between persistent firing supported by neural network with and without the CAN current had not been conducted. This left multiple possible roles of the CAN current in supporting in vivo-like persistent firing unclear.

### Comparison to experimental observations

Self-terminating persistent firing was observed in our single cell model when the value of *g_CAN* was relatively small as observed in vitro. When the *g_CAN* is sufficiently large, the influx of cations from the CAN current depolarizes the membrane strongly to support relatively high frequency of firing. During persistent firing, each spike activates the calcium current and relatively high intracellular calcium concentration is maintained to support steady CAN current activation. When the *g_CAN* is relatively low, persistent firing might still be initiated because triggering stimulation induces a relatively strong depolarization, high firing frequency, sufficient intracellular calcium, and sufficient CAN current activation. However, once stimulation is removed, current influx from the CAN current may not provide as strong depolarization, and the resulting firing frequency might be lower. This in turn lowers the intracellular calcium level and this level of calcium might not be sufficient to support the same amount of CAN current activation. In this case, the cell goes into a negative feedback loop leading to eventual termination of persistent firing. Intracellular calcium levels of a model cell that supports long-lasting persistent firing (Fig 1E(iii)) are maintained at a stable level for the duration of the persistent activity. Intracellular calcium levels in a model cell that shows self-terminating persistent firing (Fig 1F(iii)), however, slowly regress back to the baseline and persistent activity stops.

We have shown that our simple network can support persistent firing in a physiologically plausible frequency range (3–50 Hz) when the CAN current was included in the model (Figs 2 and 3). While NMDA receptor activation is required for persistent firing in vivo [71] cholinergic receptor activation is also required in similar tasks [72]. In addition, NMDA receptor blockade in the hippocampus did not impair hippocampus dependent working memory task [73]. Therefore, the NMDA component and the CAN current through cholinergic activation may both contribute to support persistent firing, and the contribution from these two mechanisms might be different depending on the areas of the brain.

We also demonstrated that persistent firing in the physiological frequency range was supported for a wide range of excitatory synaptic conductance, starting with relatively low conductance when the CAN current was present (Figs 2 and 3). Experimental studies have indicated that the cholinergic receptor activation suppresses excitatory synapses in the hippocampus [74, 75]. Therefore, the ability for the network to support persistent firing with relatively small excitatory synaptic conductance during memory tasks might be useful in vivo.

The results of our study indicated that the CAN current allows persistent activity to continue even when the firing pattern is entrained by theta-like oscillations (Fig 5). On the other hand, persistent activity was shut off by the trough of the oscillation in the pure network model. During memory tasks that require a short-term informtaion retention, elevated levels of acetylcholine are observed in the hippocampus [76–78]. At the same time, the amplitude of theta oscillations measured by intracranial EEG in humans is strongly elevated [79]. Hippocampal pyramidal cells are modulated by theta oscillations in a trace conditioning task which requires temporal information retention [58]. During theta oscillations, firing of pyramidal cells in the hippocampus are entrained by the theta oscillations [80]. Since the theta oscillations is synchronized locally, neighboring pyramidal neurons may undergo hyperpolarization phase simultaneously. As we demonstrated, this could cause termination of persistent firing supported by synaptic excitations among these cells. The ability for the CAN current to be active across multiple theta cycle may, therefore, be crucial for supporting persistent firing in the presence of theta oscillations in vivo.

The recurrent collaterals of the CA3 areas are suggested to play a major role in retrieving originally stored patterns of information given an incomplete input pattern (pattern completion) [16,17,63]. Storage of patterns by strengthening the recurrent synaptic connections underlies pattern completion in proposed models [81–83]. In the pure network model, relatively strong synaptic connections support both pattern completion and persistent firing. This might be inconsistent with the role of the hippocampus in short-term information retention. Human and animal studies indicate that the hippocampus supports short-term information retention particularly when stimuli are novel [4] and during acquisition of the task [3]. In these conditions, synaptic network may not have been strengthened yet to be able to support persistent firing.

On the other hand, the acetylcholine level may be heightened by the novelty and learning [84]. In such condition, the hybrid model can support persistent firing solely by the CAN current without the synaptic strengthening. Synaptic strengthening can be done afterward to support pattern completion. Indeed, a recent in vitro study indicated that transient receptor potential (TRPC) channels, which underlie the CAN current, support synaptic plasticity in the hippocampus [85].

### Conclusions and future directions

In conclusion, heightened cholinergic activation during memory tasks may endow the neural network with both intrinsic and synaptic mechanisms to support persistent firing. This hybrid of the CAN current and the synaptic network may be crucial for supporting a physiological and robust persistent firing in vivo.

While our small scale network model could demonstrate multiple novel insights, a larger scale network simulation with a random network structure will be beneficial. In our simulations, three pyramidal cell models were firing in synchrony due to the uniform network structure. However, Gutkin et al has pointed out that asynchronous activity could be beneficial in supporting persistent firing [86]. They further demonstrated that a brief stimulation could terminate persistent firing in their model by inducing a synchronization. Relative spike-timings between neurons may affect the termination or initiation of persistent firing even with the CAN current included. In addition, it has been reported experimentally that persistent firing in the layer III neurons in the lateral entorhinal cortex can be terminated by an excitatory input [87]. This mechanism involves the activation of BK channels which turns off the CAN current. Although such mechanism has not been reported in the hippocampus, it would be of interest to investigate these issues as additional mechanisms for controlling persistent firing in vivo.

A complementally approach for investigating the robustness and flexibility of neural networks with respect to persistent activity is to use bifurcation theory methods [88]. Although these methods are mathematically strong, it is in general difficult to apply to biological conductance based models like our current model. However, such mathematical analysis might be possible in a simplified version of our model and could strengthen our observations.

## Supporting Information

S1 Fig Effects of [Ca]c value on initiation and maintenance of persistent firing. (A and B) Responses of the model pyramidal cell to a brief current injection (500 pA, 86 ms) when [*Ca*]_*c*_ value was 0.65 μM and 0.85 μM, respectively. (C—E) Responses of the model pyramidal cell to a current stimulation (2s, 150pA) when [*Ca*]_*c*_ value was 0.65, 0.75 and 0.85 μM, respectively. Increasing the value of [*Ca*]_*c*_ caused a reduction in persistent firing frequency and eventual cessation of persistent activity.
(TIF)

S2 Fig CAN current in voltage clamp in the pyramidal cell model. (A) Current response. (B) Voltage command. A brief voltage step depolarization (-50 to 0 mV) for 10 ms induced the CAN current activation.
(TIF)
