# Correction: A Robust *In Vivo*-Like Persistent Firing Supported by a Hybrid of Intracellular and Synaptic Mechanisms

**DOI:** 10.1371/journal.pone.0130237

**Published:** 2015-06-03

**Authors:** The PLOS ONE Staff

There is an error in the first sentence of the Introduction. The correct sentence is: Behavioral studies indicate involvement of the prefrontal cortex and medial temporal lobe (MTL) during memory tasks that require short-term (200ms- 30s) information retention [1–4]. The publisher apologizes for this error.

## References

[1] Jochems A, Yoshida M (2015) A Robust *In Vivo*-Like Persistent Firing Supported by a Hybrid of Intracellular and Synaptic Mechanisms. PLoS ONE 10(4): e0123799 doi: 10.1371/journal.pone.0123799 25901969 10.1371/journal.pone.0123799 PMC4406621

