# Supplementary figures and images for: A Robust In Vivo-Like Persistent Firing Supported by a Hybrid of Intracellular and Synaptic Mechanisms
Source: PLoS One. 2015 Apr 22;10(4):e0123799. doi: 10.1371/journal.pone.0123799 (PMC4406621; doi:10.1371/journal.pone.0123799)

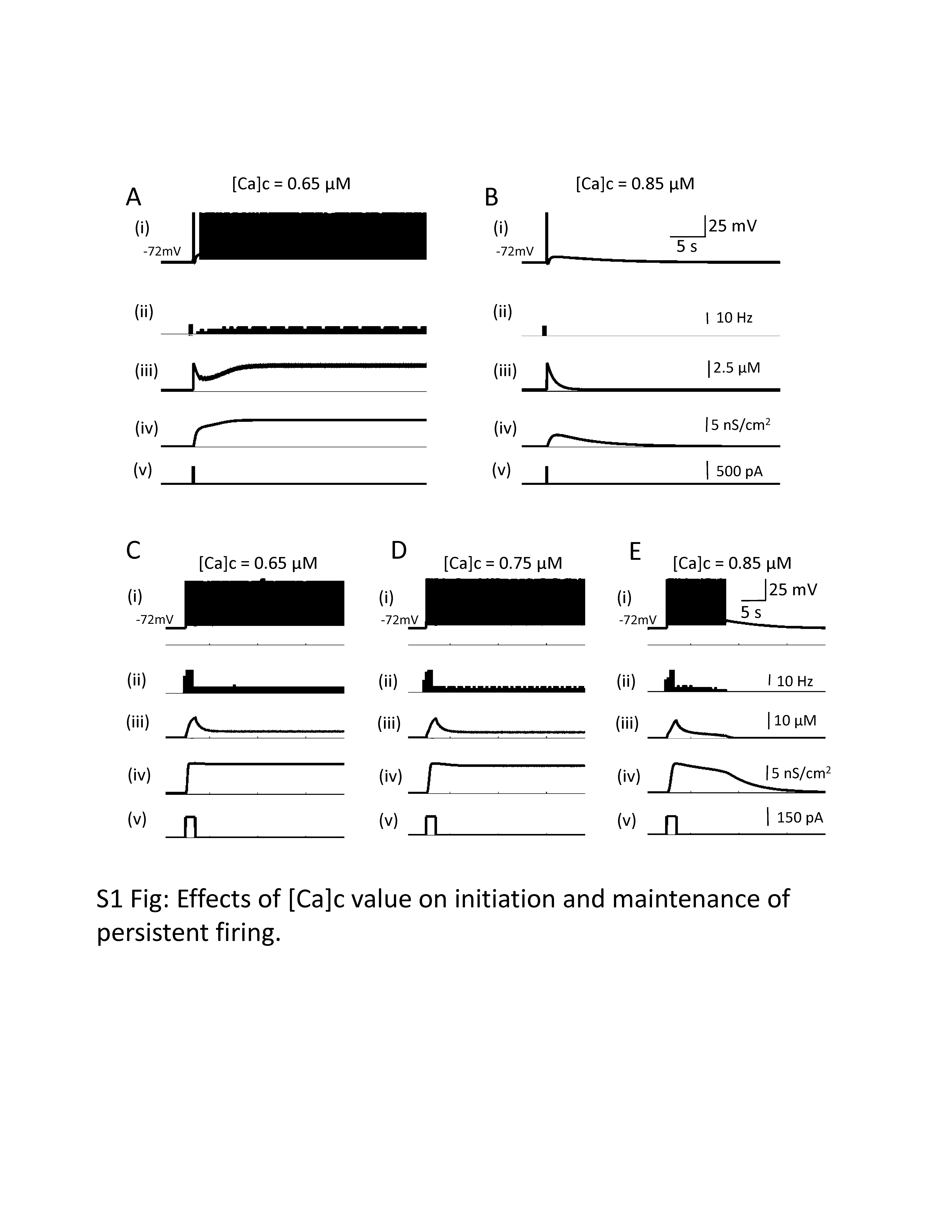

Supplement: S1 Fig — (A and B) Responses of the model pyramidal cell to a brief current injection (500 pA, 86 ms) when [Ca]c value was 0.65 μM and 0.85 μM, respectively. (C—E) Responses of the model pyramidal cell to a current stimulation (2s, 150pA) when [Ca]c value was 0.65, 0.75 and 0.85 μM, respectively. Increasing the value of [Ca]c caused a reduction in persistent firing frequency and eventual cessation of persistent activity. (TIF) [file pone.0123799.s001.tif]

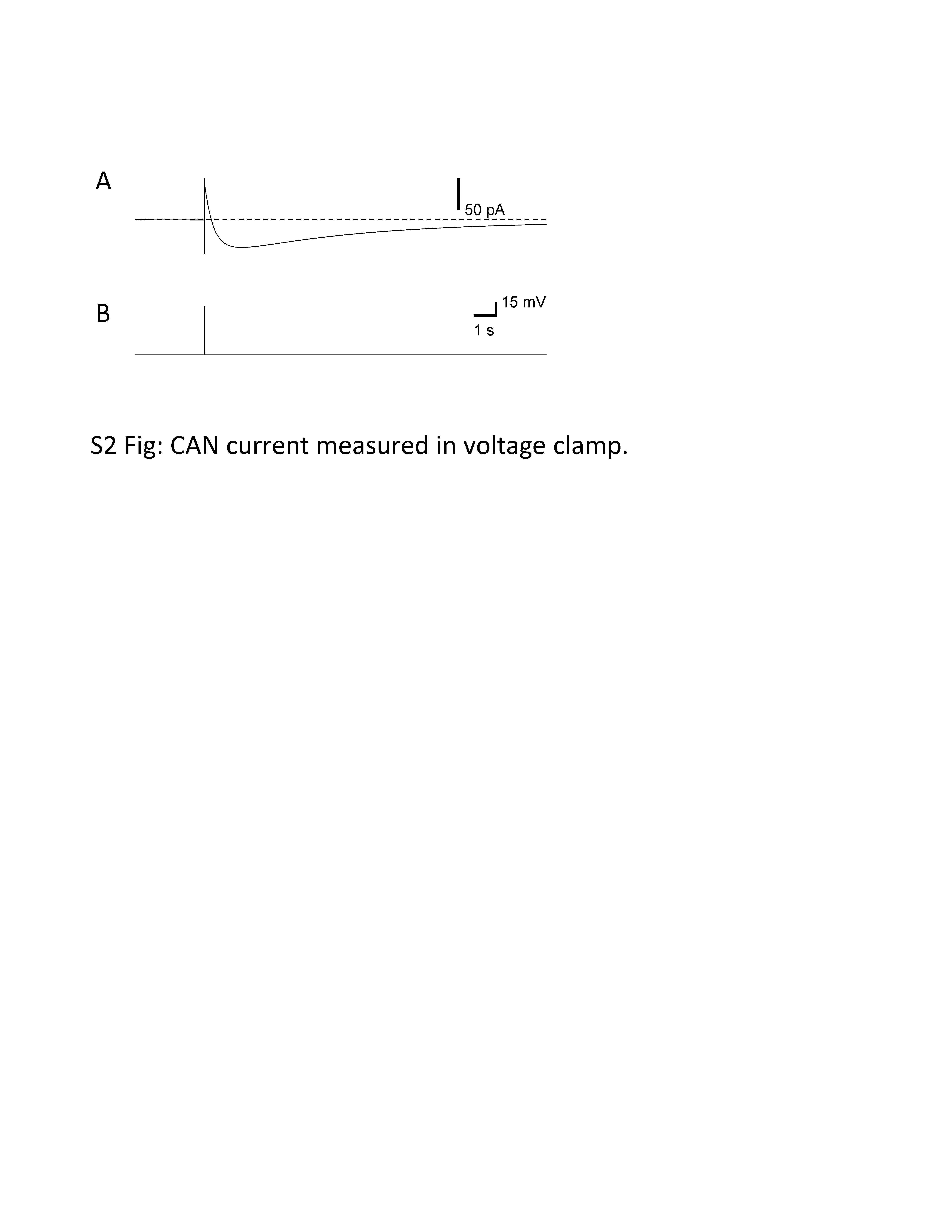

Supplement: S2 Fig — (A) Current response. (B) Voltage command. A brief voltage step depolarization (-50 to 0 mV) for 10 ms induced the CAN current activation. (TIF) [file pone.0123799.s002.tif]
